# NINJ1 oligomerises on large apoptotic cell-derived extracellular vesicles to regulate vesicle stability and cellular content release

**DOI:** 10.3389/fimmu.2025.1599809

**Published:** 2025-08-19

**Authors:** Bo Shi, Caolingzhi Tang, Stephanie F. Rutter, Omar Audi, Dilara C. Ozkocak, Alice M. Trenerry, Daniel S. Simpson, Scott A. Williams, Quan T. Le, Gemma F. Ryan, Ponsuge T. M. Cooray, James E. Vince, Jason M. Mackenzie, Mark D. Hulett, Thanh Kha Phan, Ivan K. H. Poon

**Affiliations:** ^1^ Department of Biochemistry and Chemistry, Research Centre for Extracellular Vesicles, La Trobe Institute for Molecular Science, La Trobe University, Melbourne, VIC, Australia; ^2^ Department of Microbiology and Immunology, Peter Doherty Institute for Infection and Immunity, University of Melbourne, Melbourne, VIC, Australia; ^3^ The Walter and Eliza Hall Institute of Medical Research, Melbourne, VIC, Australia; ^4^ Cancer Immunology Program, Peter MacCallum Cancer Centre, Melbourne, VIC, Australia

**Keywords:** apoptosis, apoptotic bodies, NINJ1, norovirus, inflammation, extracellular vesicles (EVs), plasma membrane rupture

## Abstract

Billions of cells undergo apoptosis, a non-inflammatory form of programmed cell death, daily as part of normal development and homeostasis. Apoptotic cells undergo apoptotic cell disassembly to release large extracellular vesicles (EVs) called apoptotic bodies (ApoBDs) to promote dead cell clearance, or otherwise proceed to an inflammatory, lytic outcome (i.e., secondary necrosis). The latter event is regulated by ninjurin-1 (NINJ1), a key executioner of plasma membrane rupture (PMR) through its oligomerisation. However, the precise role of NINJ1 at the intersection of apoptotic cell disassembly and secondary necrosis remain elusive. Here, we show that NINJ1 increasingly oligomerises upon the completion of apoptotic cell disassembly process and that higher-order NINJ1 oligomerisation occurs on ApoBDs. We also demonstrate that NINJ1 regulates PMR of ApoBDs and the release of inflammatory signals and, in part, norovirus particles. Together, our findings provide new insights into NINJ1-mediated PMR and content release-associated functions of ApoBDs.

## Introduction

Cells undergoing apoptosis, a non-inflammatory form of programmed cell death, often release ApoBDs, which are large membrane-bound EVs (1−5 μm in size). ApoBD formation is a highly co-ordinated process known as apoptotic cell disassembly consisting of three morphological steps: (i) membrane blebbing, (ii) membrane protrusion to radiate the blebs, and (iii) fragmentation of membrane protrusion leading to ApoBD release (1, 2). The disassembly of apoptotic cells to ‘bite-sized’ ApoBDs could aid the rapid cell corpses removal by phagocytes via efferocytosis, a critical process to ensure normal development and tissue homeostasis (1, 2). If phagocytes fail to eliminate cell corpses promptly, apoptosis proceeds to secondary necrosis. At this stage, dead cells undergo PMR and release autoantigens as well as proinflammatory cell contents such as danger-associated molecular patterns (DAMPs), resulting in the induction of inflammatory and autoimmune responses (3). Defects in efferocytosis and/or the persistence of DAMPs underpin the pathogenesis of chronic inflammatory and autoimmune conditions such as arthritis, systemic lupus erythematosus and Sjogren’s disease (4, 5).

It is becoming clear that ApoBDs are more than mere fragments of apoptotic cells or “garbage bags” for disposal of dead cell materials as mounting evidence demonstrates ApoBDs as key messengers that regulate cell survival and proliferation, tissue repair and immune modulation (6, 7).ApoBDs also play important roles in disease progression by releasing and/or transferring biomolecular contents and viral pathogens such as influenza A virus and avian swine flu virus (8, 9). Furthermore, various strategies to pharmacologically target ApoBD biogenesis as well as to leverage ApoBDs for drug delivery or disease therapeutics have been proposed and developed (1, 2). However, the therapeutic applications of ApoBDs are still falling short, likely due to limited insights into ApoBD biogenesis regulation and their biophysical properties, including apparent short-lived stability (about 3–6 hours in culture at 37°C (10). Therefore, defining the regulation of ApoBD stability and cellular content release is critical, not only to gain further understanding of ApoBD function and disease progression but also to extend their therapeutic potential.

Recently, NINJ1 was identified as a key executioner of PMR during secondary necrosis as well as other lytic cell death modalities, such as pyroptosis and ferroptosis (11–13). The oligomerisation of NINJ1, forming ring-like structures that cut and release membrane disks, is a key step in mediating cell lysis (13). However, precisely when NINJ1 undergo oligomerisation to mediate PMR during the progression of apoptosis is not well defined. In this study, we show that NINJ1 regulate PMR only when the dying cell has completed the apoptotic cell disassembly process and that higher-order NINJ1 oligomerisation occurs on ApoBDs. We also demonstrate that NINJ1 control ApoBD lysis and the release of DAMPs such as HMGB1. In the context of norovirus infection, NINJ1 also in part aids the release of viral particles from ApoBDs generated from infected cells. Significantly, our findings highlight the first regulatory mechanisms of EV stability and provide insights into content release-associated functions of ApoBDs.

## Results

### NINJ1 oligomerises on ApoBDs and does not affect apoptotic cell disassembly process

**Figure 1:**
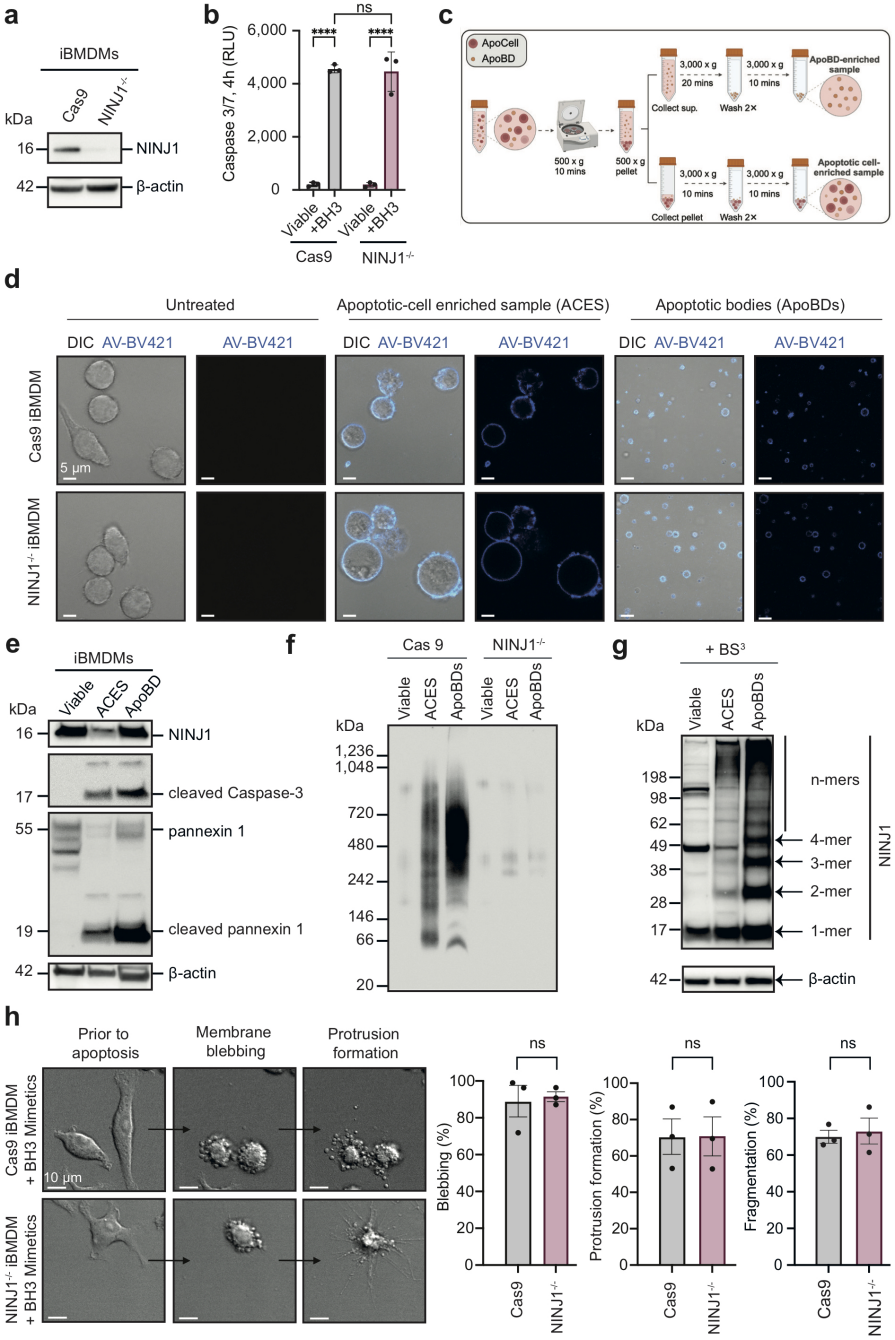
NINJ1 oligomerise on ApoBDs while does not affect apoptotic cell disassembly. **(a)** Immunoblot analysis of NINJ1 CRISPR/Cas9 targeted iBMDMs. **(b)** Caspase 3/7 activity in NINJ1^-/-^ iBMDMs and Cas9 control treated with a BH3 mimetic cocktail (2 μM ABT-737 and 10 μM S63845) for 4 h. **(c)** Schematic diagram detailing ApoBD isolation using differential centrifugation. **(d)** Confocal imaging of viable Cas9 and NINJ1^-/-^ iBMDMs or apoptotic-cell enriched sample (ACES) and ApoBDs isolated from Cas9 and NINJ1^-/-^ iBMDMs treated with a BH3 mimetics cocktail (2 μM ABT-737, 10 μM S63845) for 4 h. Annexin V (AV) was used as an indicator of apoptosis. **(e)** Detection of ApoBD markers in ApoBDs isolated from BH3 mimetic-treated iBMDMs (i.e., cleaved caspase 3 and caspase-cleaved pannexin 1), as assessed by immunoblotting. **(f)** Blue Native-PAGE and **(g)** bis(sulfosuccinimidyl) suberate (BS3) crosslinking and SDS-PAGE, followed by immunoblotting using a NINJ1 antibody to detect NINJ1 oligomerisation. **(h)** Time-lapse differential interference contrast (DIC) microscopy of BH3 mimetics treated Cas9 and NINJ1^-/-^ iBMDMs (left panel). Apoptotic cell disassembly was quantified by measuring the percentage of cells undergoing blebbing, apoptopodia formation and fragmentation (right panel). Data are mean ± S.E.M of n≥3. **(a, d–h)** show results representative of three independent experiments. Statistical significance is indicated by ***p<0.01, ns (not significant, p≥0.05), as determined by an unpaired, two-tailed Student’s t-test.

Although ApoBDs can maintain membrane integrity shortly after their formation and the onset of PMR of ApoBDs has profound implications in disease settings (2, 10), the regulation of ApoBD lysis has not been defined. Given the role of NINJ1 in PMR (11–13), we sought to determine if NINJ1 undergoes oligomerisation on ApoBDs and regulates their PMR. To this end, we first generated NINJ1-deficient immortalised bone marrow-derived macrophages (iBMDMs) using CRISPR/Cas9 gene editing (**Figure 1a**). Compared to Cas9 control, NINJ1 disruption did not significantly alter apoptosis progression as shown by Caspase 3/7 Glo^®^ assay detecting apoptotic caspase activity upon BH3 mimetics treatment (**Figure 1b**). ApoBDs were isolated from the apoptotic samples using a differential centrifugation-based approach (**Figure 1c**). We confirmed that the isolated ApoBDs are of the expected size (~1-5 μm), of high purity (~90%) and exhibit typical apoptotic markers, including phosphatidylserine exposure (indicated by annexin V staining), cleaved caspase 3 and its cleavage substrate, pannexin 1 membrane channels (**Figures 1d, e**, **Supplementary Figure 1a**). Using Blue Native-PAGE (**Figure 1f**) and bis(sulfosuccinimidyl) suberate (BS3) crosslinking followed by SDS-PAGE (**Figure 1g**), we detected a high level of NINJ1 oligomerisation on ApoBDs, compared to untreated and apoptotic cell-enriched sample (ACES). This observation not only reveals that NINJ1 could be functional on ApoBDs but also suggests that apoptotic cells complete ApoBD formation prior to transitioning to secondary necrosis, to ensure non-inflammatory cell clearance.

Consistently, by monitoring the apoptotic cell disassembly process using time-lapse microscopy, we found that both NINJ1^-/-^ and Cas9 control iBMDMs undergoing apoptosis can readily generate ApoBD through the formation of membrane blebs and membrane protrusions (**Figure 1h**). Time-course analysis of NINJ1 oligomerisation following apoptosis induction also showed limited NINJ1 oligomerisation at 4 hours post BH3 mimetics treatment (i.e. the time-point when ApoBDs were collected) (**Supplementary Figure 1b**). Collectively, these data suggest that NINJ1 does not impact ApoBD biogenesis, an important event to aid efficient removal of cell corpses via efferocytosis.

### NINJ1 controls vesicle stability, regulates DAMPs and norovirus release from ApoBDs

**Figure 2:**
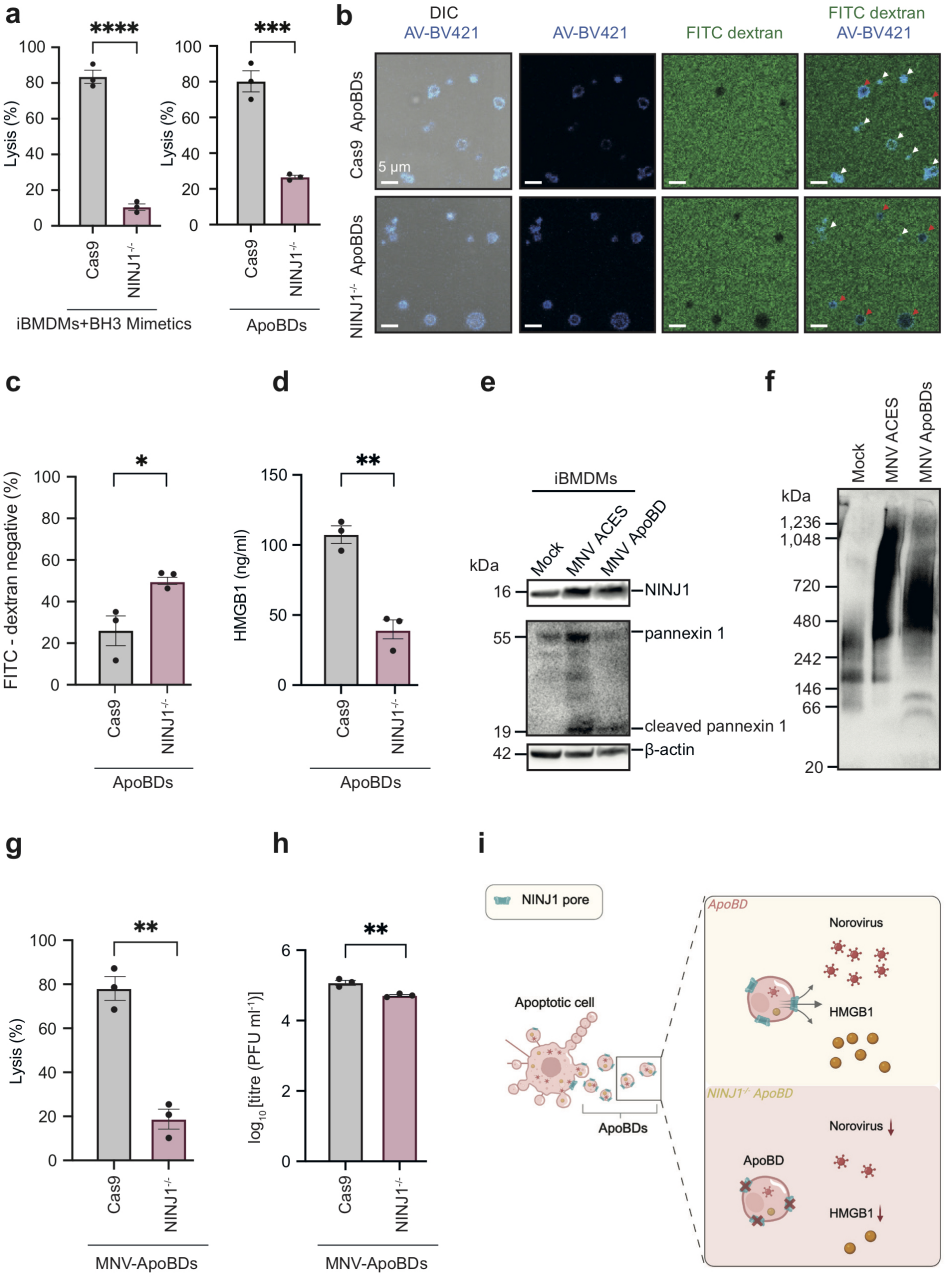
NINJ1 is a novel regulator of membrane integrity and content release from ApoBDs. **(a)** Quantification of membrane lysis of whole apoptotic sample (8 h post-apoptosis induction, left panel) and ApoBD lysis (4 h post-isolation, right panel) using the lactate dehydrogenase (LDH) release assay. **(b)** FITC-dextran (4 kDa) exclusion assay and confocal microscopy to visualise membrane lysis. Red arrows: FITC-dextran uptake, i.e., lysis. White arrows: FITC-dextran exclusion, i.e., intact. **(c)** Quantification of FITC-dextran negative in **(b)**. **(d)** Detection of HMGB1 released by ApoBDs using the Lumit^®^ HMGB1 immunoassay. **(e)** Detection of NINJ1 and ApoBD marker caspase-cleaved PANX1 in ApoBDs isolated from MNV-infected iBMDMs using immunoblotting. **(f)** Blue Native-PAGE and immunoblotting to detect oligomeric NINJ1 associated with ApoBDs derived from murine norovirus (MNV)-infected iBMDMs (17 h post-infection). **(g)** Quantification of ApoBD (4 h post-isolation) membrane lysis as measured using the LDH release assay. **(h)** Quantification of virion release from ApoBDs (4 h post-isolation) using a plaque assay. **(i)** Schematic diagram illustrating a NINJ1 oligomerisation-dependent mechanism of ApoBD membrane lysis as well as the release of damage signals/infectious agents from ApoBDs. Data are mean ± S.E.M of n≥3. Panels **(a–g)** show results representative of three independent experiments. Statistical significance is indicated by *p<0.05, **p<0.01, ***p<0.001 and ****p<0.0001, as determined by an unpaired, two-tailed Student’s t-test.

As NINJ1 oligomerises on ApoBDs, we next sought to detect PMR in ApoBDs by using two experimental approaches. First, using the lactate dehydrogenase (LDH) release assay, a standard measure of late-stage PMR (12, 13), we found that NINJ1 deficiency markedly rescued PMR, not only in whole apoptotic iBMDMs but also in ApoBD samples (**Figure 2a**). In addition, we also performed FITC-dextran exclusion assays to visualise PMR at the single vesicle level. NINJ1^-/-^ iBMDM-derived ApoBDs showed twice as much FITC-dextran exclusion as compared to Cas9 control iBMDM-derived ApoBDs, further indicating reduced PMR of NINJ1 deficient ApoBDs (**Figures 2b, c**).

NINJ1 controls important downstream consequences of cell death by facilitating PMR and the non-selective release of cellular contents, such as DAMPs and viruses from dying cells (11–14). Notably, although ApoBDs can harbour a variety of cellular contents, whether DAMPs are released from ApoBDs and the mechanism of release has not been defined. To determine whether NINJ1 is involved in DAMP release upon ApoBD lysis, we sought to detect HMGB1, a prominent DAMP, in supernatants of Cas9 and NINJ1^-/-^ ApoBDs at 4 h post-isolation using the Lumit^®^ HMGB1 bioluminescence assay. First, we found that HMGB1 are released by ApoBDs (**Figure 2d**), under conditions whereby ~80% of ApoBDs had undergone PMR (**Figure 2a**, right panel). Furthermore, the release of HMGB1 by ApoBDs was significantly impaired upon NINJ1 deficiency. In addition, viral infections that induce apoptosis can hijack resultant ApoBDs to disseminate to other bystander cells (14), with ApoBD lysis as a potential mechanism for viral release. Using mouse norovirus (MNV) as an apoptosis-inducing viral model for iBMDMs (15), we were able to isolate ApoBDs from the infected cells (**Figure 2e**) and detect NINJ1 oligomerisation on ApoBDs by Blue Native-PAGE (**Figure 2f**). We also observed a marked decrease in LDH release (**Figure 2g**) and a partial reduction (~2.5 fold decrease) of viral release from ApoBDs derived from MNV-infected NINJ1^-/-^ iBMDMs as compared to MNV-infected Cas9 control iBMDMs (**Figure 2h**).

Together, these data suggest that NINJ1 further oligomerises on ApoBDs post-biogenesis and regulates their stability and thus the release of DAMPs and viral particles (**Figure 2i**). To our knowledge, NINJ1 represents the first regulator of EV stability.

## Discussion

Our findings collectively demonstrate the timing of NINJ1 oligomerisation and its role in regulating vesicle stability and content release (including DAMPs and virions) from ApoBDs, thus providing further insights into the biology of cell death and EVs.

We extend upon recent NINJ1 discoveries by describing their ability to oligomerise at a later stage of apoptosis, particularly on ApoBDs. Downstream of apoptosis, dying cells undergo apoptotic cell disassembly and inflammatory secondary necrosis if efferocytosis fails (1, 2). As aforementioned, apoptotic cell disassembly is generally regulated by three sequential morphological steps to generate ApoBDs to facilitate dead cell clearance and intercellular communications. From the point of view of efferocytosis, it is logical and desirable that the onset of secondary necrosis, regulated by NINJ1-mediated PMR, occur post-ApoBD biogenesis as supported by our data. In fact, apoptotic cells override lytic cell death modalities as cleavage of gasdermin D by caspase 3 results in non-membrane lytic form (16). Concurrently, apoptotic cells also release ‘find-me’ signals and expose ‘eat-me’ signals to promote cell clearance by phagocytes to limit secondary necrosis (17, 18). Similar to our NIN1 findings, gasdermin E, which is activated by caspase 3 and mediates secondary necrosis, does not function as a negative regulator of apoptotic cell disassembly, notably in cell types such as T cells and monocytes (19).

To ensure immune tolerant clearance and aid intercellular communications, ApoBDs contain a range of cellular contents and biomolecules. However, as we reported previously (10), ApoBDs undergo lysis within several hours in cell culture conditions. Here, we demonstrate that NINJ1, through oligomerisation, regulates PMR and, thus, stability of ApoBDs. Despite significant effort has been made by EV researchers to study and improve EV stability for biomedical applications, NINJ1 is the first known regulator of EV stability. Consequently, NINJ1 controls the release of DAMPs such as HMGB1 and, to a certain extent, viral particles from ApoBDs. Notably, as ApoBDs can also harbour autoantigens such as DNA and histones (20, 21), regulated ApoBD lysis though NINJ1 may play a role in the progression of autoimmune diseases. Furthermore, these findings support the notion that NINJ1 is a universal PMR regulator and implicated in various chronic inflammatory and infection settings. For instance, NINJ1-dependent secretome from cells undergoing ferroptosis, an iron-dependent lytic cell death, contain mostly cytosolic or nuclear DAMP proteins including HMGB1, several histones, actin-related proteins, and heat-shock proteins. By contrast, NINJ1-independent secretome is smaller in size and abundance, and more associated with the specific biological functions (such as cytokines), and/or extracellular milieu and cell surface, instead of intracellular compartments. In addition, recent study by Wang et al. also demonstrated that NINJ1 is required for norovirus egress downstream of non-apoptotic cell death induced by viral MLKL-like protein (14). Our data therefore suggest another plausible mechanism for norovirus release via ApoBD lysis whilst consistently highlighting the significant role of NINJ1 in mediating the PMR of ApoBDs. It is worth noting that the precise mechanism of norovirus release is not fully defined and can involve non-lytic egress mechanisms such as through exosome-mediated viral transfer (22), which are likely to be independent of NINJ1.

Our findings also highlight the possibilities of repurposing NINJ1-targeting therapeutics, such as NINJ1 blocking antibodies (23), that are currently being developed for ApoBD-associated disease settings. Furthermore, as the applicability of ApoBDs as therapeutic agents (e.g., vaccine development, immunotherapies, regenerative therapies, drug delivery platforms) and diagnostic tools has been largely limited by vesicle instability, the NINJ1-targeting strategies to control ApoBD stability would greatly improve its therapeutic and diagnostic potential.

## Materials and methods

### Reagents

BH3 mimetics ABT-737 (targets Bcl-2, Bcl-xl and Bcl-w) and S63845 (targets Mcl-1) were purchased from MedChemExpress (Monmouth Junction, NJ, USA). Fluorescently-labelled Annexin V (AV) and AV binding buffer were from BD Biosciences. TO-PRO-3 was purchased from Thermo-Fisher Scientific (Carlsbad, CA, USA).

### Cell culture

Cas9 control and NINJ1^−/−^ murine immortalised bone marrow-derived macrophages (iBMDMs) were generated as previously reported (24) and cultured in complete Gibco^®^ Dulbecco’s Modified Eagle Medium media, 1 g/L glucose (Thermo-Fisher Scientific), supplemented with 10% (v/v) foetal bovine serum (FCS; Scientifix, Australia), 50 IU/ml penicillin, 50 µg/ml streptomycin (Thermo-Fisher Scientific), and 0.2% (v/v) MycoZap (Lonza, Switzerland) at 37°C in a humidified atmosphere with 5% CO_2_.

### Induction of apoptosis

Cas9 or NINJ1^-/-^ iBMDMs were seeded in T175 flasks at a density of 5 × 10^6^ cells per flask. On the next day, cells were treated with 2 µM of ABT-737 and 10 µM of S63845 in serum-free DMEM media supplemented with 1% bovine serum albumin (Sigma-Aldrich, St. Louis, MO, USA) for 4 h.

### Caspase-Glo 3/7 assay

Caspase 3/7 activity was measured using the Caspase-Glo 3/7 Glo^®^ assay (Promega, WI, US) according to the manufacturer’s instructions.

### Apoptotic body purification

ApoBDs were isolated from BH3 mimetic-treated or MNV-infected iBMDMs using differential centrifugation as previously described (25). Briefly, cells were centrifuged at 500 x g for 10 min to remove whole apoptotic cells. The supernatant was collected and centrifuged at 3,000 × *g* for 20 min to pellet ApoBDs. After three washes with 1× PBS (Thermo-Fisher), isolated ApoBDs were quality controlled by flow cytometry with AV (1:100) as well as TO-PRO-3 (0.5 µM) staining and counted with AccuCount blank flow cytometry particles (ProSciTech) before proceeding to further assays. Flow cytometry was performed using a BD FACSCanto II flow cytometer (BD Bioscience). Data were analysed using FlowJo software v10.10.0, with the gating strategy described by Jiang et al. (2016) (26).

### Confocal microscopy

Apoptotic cell-enriched sample (ACES) and ApoBDs were harvested and stained with A5-BV421 (1:100) prior to seeding on an 8-well chambered NuncTM Lab-TekTM II cover glass (Nunc, Denmark). Images were captured using a Zeiss 800/900 confocal laser scanning microscope (Zeiss, Germany) equipped with a 63x magnification oil immersion lens, at 37°C and 5% CO_2_. Image analysis was performed using Zen software v2.6.

### Blue native polyacrylamide gel electrophoresis (Blue Native-PAGE)

ACES and ApoBDs were prepared from BH3 mimetic-treated iBMDMs. Untreated cells, included as a viable control, were detached using an Accutase solution (Sigma-Aldrich). All samples were lysed using a native-PAGE lysis buffer containing 150 mM NaCl, 1% Digitonin, 50 mM Tris (pH 7.5), and a protease inhibitor cocktail (Roche) at 4°C for 30 min. NativePAGE sample buffer and Coomassie G-250 (Thermo-Fisher) were then added to the lysates before electrophoresis using a Native-PAGE 3-12% gel (Thermo-Fisher). Immunoblotting was performed subsequently.

### Crosslinking assays and SDS-PAGE

Viable, ACES, ApoBD samples were resuspended in 1× PBS containing 3 mM bis(sulfosuccinimidyl) suberate (BS3) crosslinker (Thermo-Fisher) and incubated for 5 min at room temperature. The reaction was stopped by adding 20 mM Tris (pH 7.5), incubated for 15 min at room temperature and then membrane washed with 1× PBS at 3,000 x g for 10 minutes. ApoBDs/cell lysates were obtained by adding chilled lysis buffer containing 1% IGEPAL^®^ CA-630, 20 mM HEPES (pH 7.4), 10% glycerol, 1% Triton X-100, 150 mM NaCl, 50 mM NaF and Roche protease inhibitor cocktail. Proteins were resolved using NuPAGE 4-12% Bis-Tris gel (Invitrogen). Immunoblotting was performed afterwards.

### Immunoblotting

After gel electrophoresis, proteins were transferred onto a PVDF membrane. Immunoblotting was performed with the following primary antibodies: NINJ1 Polyclonal Antibody (1:1000, Thermo-Fisher, PA5-95755), anti-pannexin 1 (1:1000; Cell Signalling), anti-caspase 3 (1:1000; Cell Signalling), and anti-β-actin (1:4000, clone AC-74, Sigma-Aldrich, A2228) in 3% BSA in PBS-T (i.e., 1× PBS with 0.1% Tween-20). Following the primary antibody incubation, the blots were incubated with HRP-conjugated secondary antibodies: sheep anti-mouse (1:5000, Millennium Science) or donkey anti-rabbit (1:5000, Millennium Science) in 5% milk in PBST (0.1% Tween-20). Chemiluminescence detection of target proteins were done using ECL Prime reagent (Bio-strategy) with a Syngene G:BOX gel documentation system (Syngene).

### Time-lapse differential interference contrast microscopy

Cas9 and NINJ1^-/-^ iBMDMs were seeded in an 8-well Nunc^®^ Lab-Tek^®^ II chamber slide overnight. On the following day, BH3 mimetics were added and cells were imaged using a Zeiss spinning disk confocal microscope (Zeiss, Germany) equipped with a 63× oil objective, at 37°C with 5% CO_2_. Images were captured every 2 minutes over a 12-hour period and analysed using Zen Blue imaging software (Zeiss, Germany).

### Lactate dehydrogenase release assay

Cas9 or NINJ1^-/-^ iBMDMs were treated with BH3 mimetics in a 96-well plate containing serum-free DMEM media supplemented with 1% BSA in a humidified atmosphere at 37°C, 5% CO_2_ for 8 hours. For ApoBD samples, 6×10^5^ ApoBDs (isolated at 4 h post-apoptosis induction) were seeded per well in a 96-well plate for 4 h. Subsequently, supernatants were then harvested and centrifuged at 500 × g for 20 min to remove cells and ApoBDs. LDH was then detected using a LDH Cytotoxicity Assay Kit (Abcam) as per the manufacturer’s instructions. Absorbance was measured at 450 nm using a SpectraMax M5e Plate Reader (Molecular Devices, CA), and data were analysed using SoftMax Pro 5.2 software (Molecular Devices).

### FITC-dextran uptake assay

A total of 3×10^5^ ApoBDs were seeded in an 8-well Nunc^®^ Lab-Tek^®^ II chamber slide pre-treated with poly-L-lysine and incubated at 37°C with 5% CO_2_ in a humidified atmosphere. After 1 h, 100 μg/ml FITC-dextran (4 kDa, Sigma-Aldrich) and A5-BV421 (1:100 dilution) were added. Images were captured using confocal microscopy with a Zeiss 800/900 Confocal Laser Scanning Microscope (Zeiss, Germany) and analysed using Zen software version 2.6. To quantify the percentage of FITC-negative ApoBDs, the number of intact vesicles (determined by the absence of FITC-dextran staining) in a 93.8 μM^2^ area was divided by the total number of ApoBDs in the same area. In total, 922 and 1,269 ApoBDs from Cas9 and NINJ1^-/-^ iBMDMs, respectively, were quantified from 12 tile regions for both cell lines across three independent experiments.

### HMGB1 detection assay

ApoBD supernatants were harvested as aforementioned for the LDH release assay. HMGB1 was then detected using a Lumit^®^ HMGB1 Human/Mouse Immunoassay kit (Promega) as per the manufacturer’s instructions.

### Mouse norovirus infection

A total of 3.5 × 10^6^ Cas9 iBMDMs or NINJ1^-/-^ iBMDMs were seeded in a T175 flask for 24 h. Cells were infected with the MNV CW1 strain at an MOI of 5, as previously described (15). Briefly, the spent media was removed and replaced with 6 mL of serum-free DMEM containing MNV, allowing for a 1 h absorption at 37°C. Subsequently, 20 mL of infection media (DMEM supplemented with 2% FCS, 110 mg/L sodium pyruvate, 2 mM GlutaMAX) was added and cells incubated for 17 h at 37°C with 5% CO_2_.

### Plaque assay

ApoBDs from MNV-infected iBMDMs and resultant ApoBD supernatants were prepared and collected as aforementioned. The ApoBD supernatants were stored at −80°C prior to the plaque assay. One day before the assay, RAW264.7 cells were seeded to approximately 70% confluency in a 12-well plate. Six ten-fold serial dilutions of ApoBD supernatants (10^−2^ to 10^−7^) in DMEM were added onto the RAW264.7 cells for 1 h. After infection, the inoculum was removed and an overlayer containing 70% DMEM, 2.4% FCS, 13.3 mM NaHCO3, 24.4 mM HEPES, 200 mM GlutaMAX, and 0.35% low melt-point agarose was added. Samples were incubated at 4°C for 30 min to allow the agarose to solidify before transferring to a humidified, 37°C, 5% CO_2_ incubator for 48 h. Cells were then fixed with 10% formalin for 1 h at room temperature. Plaques were visualised and counted after staining with toluidine blue.

### Quantification and statistical analysis

Data are presented as means ± SEM. Statistical analyses were performed using unpaired, two-tailed Student’s t-test.

## Data Availability

The original contributions presented in the study are included in the article/**Supplementary Material**. Further inquiries can be directed to the corresponding authors.

## Supplementary material

Supplementary Figure 1 Validation of ApoBD purity and time-course analysis of NINJ1 oligomerisation following apoptosis induction. **(a)** Purity assessment and quality control for ApoBDs isolated from BH3 mimetic-treated iBMDMs. **(b)** Blue Native-PAGE followed by immunoblotting using a NINJ1 antibody to detect NINJ1 oligomerisation at 0, 2, 4 and 10 hours post apoptosis induction by BH3 mimetics cocktail (2 μM ABT-737, 10 μM S63845).

## References

[1] Phan TK Ozkocak DC Poon IKH. Unleashing the therapeutic potential of apoptotic bodies. Biochem Soc Trans. (2020) 48:2079–88. doi: 10.1042/BST20200225, PMID: 32869835 PMC7609033

[2] Shi B Phan TK Poon IKH. Extracellular vesicles from the dead: the final message. Trends Cell Biol. (2025) 35:439–52. doi: 10.1016/j.tcb.2024.09.005, PMID: 39438206

[3] Sachet M Liang YY Oehler R. The immune response to secondary necrotic cells. Apoptosis. (2017) 22:1189–204. doi: 10.1007/s10495-017-1413-z, PMID: 28861714 PMC5630647

[4] Xing J Wang K Xu YC Pei ZJ Yu QX Liu XY. Efferocytosis: Unveiling its potential in autoimmune disease and treatment strategies. Autoimmun Rev. (2024) 23:103578. doi: 10.1016/j.autrev.2024.103578, PMID: 39004157

[5] Mehrotra P Ravichandran KS. Drugging the efferocytosis process: concepts and opportunities. Nat Rev Drug Discovery. (2022) 21:601–20. doi: 10.1038/s41573-022-00470-y, PMID: 35650427 PMC9157040

[6] Brock CK Wallin ST Ruiz OE Samms KM Mandal A Sumner EA. Stem cell proliferation is induced by apoptotic bodies from dying cells during epithelial tissue maintenance. Nat Commun. (2019) 10:1044. doi: 10.1038/s41467-019-09010-6, PMID: 30837472 PMC6400930

[7] Ma Q Liang M Wu Y Ding N Duan L Yu T. Mature osteoclast–derived apoptotic bodies promote osteogenic differentiation via RANKL-mediated reverse signaling. J Biol Chem. (2019) 294:11240–7. doi: 10.1074/jbc.RA119.007625, PMID: 31167789 PMC6643026

[8] Gao P Zhou L Wu J Weng W Wang H Ye M. Riding apoptotic bodies for cell–cell transmission by African swine fever virus. Proc Natl Acad Sci. (2023) 120:e2309506120. doi: 10.1073/pnas.2309506120, PMID: 37983498 PMC10691326

[9] Atkin-Smith GK Duan M Zanker DJ Loh L Nguyen THO Koutsakos M. Monocyte apoptotic bodies are vehicles for influenza A virus propagation. Commun Biol. (2020) 3:223. doi: 10.1038/s42003-020-0955-8, PMID: 32385344 PMC7210108

[10] Poon IKH Parkes MAF Jiang L Atkin-Smith GK Tixeira R Gregory CD. Moving beyond size and phosphatidylserine exposure: evidence for a diversity of apoptotic cell-derived extracellular vesicles *in vitro* . J Extracell Vesicles. (2019) 8:1608786. doi: 10.1080/20013078.2019.1608786, PMID: 31069027 PMC6493268

[11] Ramos S Hartenian E Santos JC Walch P Broz P. NINJ1 induces plasma membrane rupture and release of damage-associated molecular pattern molecules during ferroptosis. EMBO J. (2024) 43:1164–86. doi: 10.1038/s44318-024-00055-y, PMID: 38396301 PMC10987646

[12] Kayagaki N Kornfeld OS Lee BL Stowe IB O’Rourke K Li Q. NINJ1 mediates plasma membrane rupture during lytic cell death. Nature. (2021) 591:131–6. doi: 10.1038/s41586-021-03218-7, PMID: 33472215

[13] David L Borges JP Hollingsworth LR Volchuk A Jansen I Garlick E. NINJ1 mediates plasma membrane rupture by cutting and releasing membrane disks. Cell. (2024) 187:2224–2235.e16. doi: 10.1016/j.cell.2024.03.008, PMID: 38614101 PMC11055670

[14] Wang G Zhang D Orchard RC Hancks DC Reese TA. Norovirus MLKL-like protein initiates cell death to induce viral egress. Nature. (2023) 616:152–8. doi: 10.1038/s41586-023-05851-w, PMID: 36991121 PMC10348409

[15] Deerain JM Aktepe TE Trenerry AM Ebert G Hyde JL Charry K. Murine norovirus infection of macrophages induces intrinsic apoptosis as the major form of programmed cell death. Virology. (2024) 589:109921. doi: 10.1016/j.virol.2023.109921, PMID: 37939648

[16] Poreba M Salvesen G. Return of the ice age: caspases safeguard against inflammatory cell death. Cell Chem Biol. (2017) 24:550–2. doi: 10.1016/j.chembiol.2017.05.001, PMID: 28525771

[17] Fond AM Ravichandran KS. Clearance of dying cells by phagocytes: mechanisms and implications for disease pathogenesis. Adv Exp Med Biol. (2016) 930:25–49. doi: 10.1007/978-3-319-39406-0_2, PMID: 27558816 PMC6721615

[18] Fond AM Ravichandran KS. Clearance of Dying Cells by Phagocytes: Mechanisms and Implications for Disease Pathogenesis. In: Gregory CD, editor. Apoptosis in Cancer Pathogenesis and Anti-cancer Therapy: New Perspectives and Opportunities. Springer International Publishing, Cham (2016). p. 25–49. doi: 10.1007/978-3-319-39406-0_2, PMID: PMC6721615 27558816

[19] Tixeira R Shi B Parkes MAF Hodge AL Caruso S Hulett MD. Gasdermin E does not limit apoptotic cell disassembly by promoting early onset of secondary necrosis in jurkat T cells and THP-1 monocytes. Front Immunol. (2018) 9:2842. doi: 10.3389/fimmu.2018.02842, PMID: 30564238 PMC6288192

[20] Jiang L Paone S Caruso S Atkin-Smith GK Phan TK Hulett MD. Determining the contents and cell origins of apoptotic bodies by flow cytometry. Sci Rep. (2017) 7:14444. doi: 10.1038/s41598-017-14305-z, PMID: 29089562 PMC5663759

[21] Berda-Haddad Y Robert S Salers P Zekraoui L Farnarier C Dinarello CA. Sterile inflammation of endothelial cell-derived apoptotic bodies is mediated by interleukin-1α. Proc Natl Acad Sci. (2011) 108:20684–9. doi: 10.1073/pnas.1116848108, PMID: 22143786 PMC3251090

[22] Todd KV Tripp RA. Exosome-mediated human norovirus infection. PloS One. (2020) 15:e0237044. doi: 10.1371/journal.pone.0237044, PMID: 32745122 PMC7398508

[23] Shen J Chen R Duan S. NINJ1: Bridging lytic cell death and inflammation therapy. Cell Death Disease. (2024) 15:831. doi: 10.1038/s41419-024-07203-6, PMID: 39543132 PMC11564778

[24] Simpson DS Pang J Weir A Kong IY Fritsch M Rashidi M. Interferon-γ primes macrophages for pathogen ligand-induced killing via a caspase-8 and mitochondrial cell death pathway. Immunity. (2022) 55:423–441.e9. doi: 10.1016/j.immuni.2022.01.003, PMID: 35139355 PMC8822620

[25] Phan TK Poon IK Atkin-Smith GK. Detection and isolation of apoptotic bodies to high purity. JoVE (Journal Visualized Experiments). (2018) 138):e58317. doi: 10.3791/58317, PMID: 30148494 PMC6126795

[26] Jiang L Tixeira R Caruso S Atkin-Smith GK Baxter AA Paone S. Monitoring the progression of cell death and the disassembly of dying cells by flow cytometry. Nat Protoc. (2016) 11:655–63. doi: 10.1038/nprot.2016.028, PMID: 26938116

