# Supplementary figures and images for: NINJ1 oligomerises on large apoptotic cell-derived extracellular vesicles to regulate vesicle stability and cellular content release
Source: Front Immunol. 2025 Aug 19;16:1599809. doi: 10.3389/fimmu.2025.1599809 (PMC12401683; doi:10.3389/fimmu.2025.1599809)

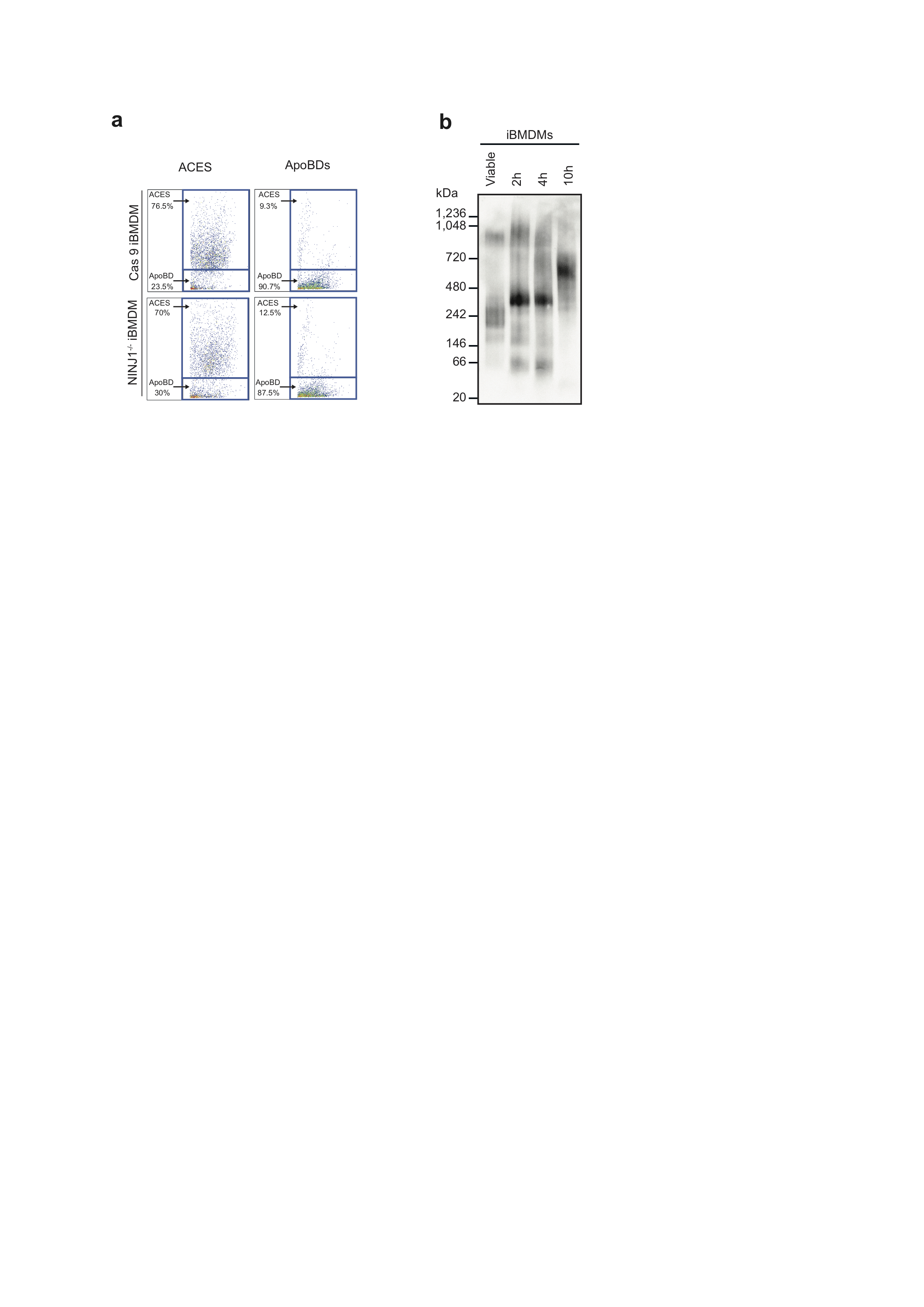

Supplement: Supplementary Figure 1 — Validation of ApoBD purity and time-course analysis of NINJ1 oligomerisation following apoptosis induction. (a) Purity assessment and quality control for ApoBDs isolated from BH3 mimetic-treated iBMDMs. (b) Blue Native-PAGE followed by immunoblotting using a NINJ1 antibody to detect NINJ1 oligomerisation at 0, 2, 4 and 10 hours post apoptosis induction by BH3 mimetics cocktail (2 μM ABT-737, 10 μM S63845). [file Image1.jpeg]
